# A rare case of Kager's fat pad syndrome with retrocalcaneal bursitis following ankle sprain

**DOI:** 10.11604/pamj.2026.53.108.48836

**Published:** 2026-03-02

**Authors:** Swapnil Ramteke, Palash Satone

**Affiliations:** 1 Department of Sports Physiotherapy, Ravi Nair Physiotherapy College, Datta Meghe Institute of Higher Education and Research, Sawangi (Meghe), Wardha, Maharashtra, India

**Keywords:** Kager's fat pad, ankle sprain, retrocalcaneal bursitis, rehabilitation

## Image in medicine

The Kager´s fat pad is an adipose structure located between the Achilles tendon, calcaneus, and flexor hallucis longus. Kager´s fat pad syndrome results from overuse, trauma, and underlying conditions like tendonitis. Pain and inflammation at the posterior side of the ankle are the primary complaints of patients with Kager´s fat pad syndrome. A 34-year-old male monk presented with posterior ankle pain, localized swelling, and reduced range of motion (ROM) that developed over several weeks. He had a history of barefoot walking and an ankle sprain sustained while climbing stairs two months earlier. Clinical examination revealed posterior ankle tenderness and dorsiflexion limitation. Magnetic resonance imaging (MRI) was ordered to confirm suspicions of ligamentous or soft tissue injury. Imaging demonstrated edema of Kager´s fat pad, sprain of the anterior and posterior talofibular ligaments, sprain of the calcaneofibular ligament, and retrocalcaneal bursitis. Kager´s fat pad pathology is a recognized but underdiagnosed source of posterior ankle pain, often seen in conjunction with other mechanical stressors. Imaging findings guided a conservative treatment plan consisting of cryotherapy, shockwave therapy, Maitland mobilization, taping, myofascial release, range of motion exercises, and strengthening exercises. The patient showed marked improvements in pain and ankle function over four weeks. This case supports prior findings that musculoskeletal imaging significantly enhances physiotherapy treatment planning.

**Figure 1:**
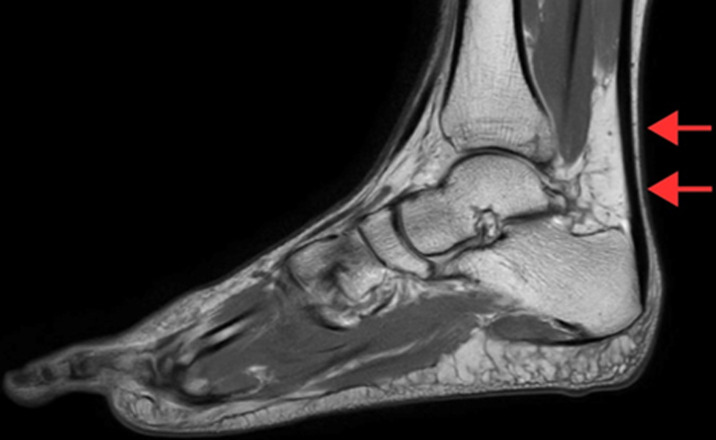
sagittal T2-weighted MRI highlighting Kager’s fat pad syndrome (arrows) and associated retrocalcaneal bursitis

